# Interplay Between Intracellular Transport Dynamics and Liquid‒Liquid Phase Separation

**DOI:** 10.1002/advs.202308338

**Published:** 2024-03-06

**Authors:** Ming‐Li Zhang, Ziheng Zhang, Xue‐Zhi Niu, Hui‐Ying Ti, Yu‐Xuan Zhou, Bo Gao, Yiwei Li, Ji‐Long Liu, Xiaosong Chen, Hui Li

**Affiliations:** ^1^ School of Systems Science and Institute of Nonequilibrium Systems Beijing Normal University Beijing 100875 China; ^2^ School of Life Science and Technology ShanghaiTech University Shanghai 201210 China; ^3^ The Key Laboratory for Biomedical Photonics of MOE at Wuhan National Laboratory for Optoelectronics – Hubei Bioinformatics and Molecular Imaging Key Laboratory Department of Biomedical Engineering College of Life Science and Technology Huazhong University of Science and Technology Wuhan 430074 China

**Keywords:** diffusion, intracellular dynamics, liquid‒liquid phase separation, molecular crowding

## Abstract

Liquid‒liquid phase separation (LLPS) is a ubiquitous process in which proteins, RNA, and biomolecules assemble into membrane‐less compartments, playing important roles in many biological functions and diseases. The current knowledge on the biophysical and biochemical principles of LLPS is largely from in vitro studies; however, the physiological environment in living cells is complex and not at equilibrium. The characteristics of intracellular dynamics and their roles in physiological LLPS remain to be resolved. Here, by using single‐particle tracking of quantum dots and dynamic monitoring of the formation of stress granules (SGs) in single cells, the spatiotemporal dynamics of intracellular transport in cells undergoing LLPS are quantified. It is shown that intracellular diffusion and active transport are both reduced. Furthermore, the formation of SG droplets contributes to increased spatial heterogeneity within the cell. More importantly, the study demonstrated that the LLPS of SGs can be regulated by intracellular dynamics in two stages: the reduced intracellular diffusion promotes SG assembly and the microtubule‐associated transport facilitates SG coalescences. The work on intracellular dynamics not only improves the understanding of the mechanism of physiology phase separations occurring in nonequilibrium environments but also reveals an interplay between intracellular dynamics and LLPS.

## Graphical abstract

Live‐cell single‐particle tracking of quantum dots reveals the complex interplay between nonequilibrium dynamics and liquid–liquid phase separation (LLPS). Intracellular diffusion and active transport are reduced after LLPS, due to increased molecular crowding and heterogeneity of intracellular environments. Additionally, LLPS is regulated by intracellular dynamics: the reduced intracellular diffusion promotes stress granule (SG) assembly, and microtubule‐associated transport facilitates SG coalescence.

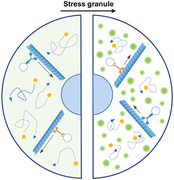

## 1 Introduction

Liquid‒liquid phase separation (LLPS) is an important cellular process that controls various biological functions, in which membrane‐less organelles (MLOs) are generated from the assembly of proteins and RNA.^[^ ^1^ ^]^ Abnormal LLPS has been linked to diseases such as cancer and neurodegenerative diseases.^[^ ^1^, ^2^ ^]^ Cellular LLPS is also a response and adaptation to external stimuli, including oxidative stress and osmotic stress. Stress granules (SGs) are a typical kind of MLO formed under stresses, with sizes ranging from 0.1 to 2 µm.^[^ ^3^ ^]^ They are mainly composed of 40S ribosomal subunits, translation initiation factors, mRNAs, RNA‐binding proteins (RBPs), and other proteins.^[^ ^4^ ^]^ In the past decade, with the active investigations of in vitro LLPS experiments, the regulatory mechanisms of phase separation have gradually been discovered, including the molecule concentration and composition, as well as environmental factors such as crowding and temperature.^[^ ^5^ ^]^ Nevertheless, as the physiological environment in cells is more complex and far from the liquid in the tubes, our understanding of in vivo LLPS inside living cells is still lacking, especially for the intracellular physical characteristics and their relationship with LLPS. For example, molecular crowding, as one of the critical factors for in vitro LLPS, has recently been brought into focus in the context of living cells since it has been shown that the extent and component of intracellular crowding are quite different from in vitro mimicking conditions.^[^ ^6^ ^]^ At present, the major accepted mechanism of molecule crowding on LLPS is the increased molecule concentrations due to the occupancy of intracellular spaces. However, the study of intracellular transport dynamics, which are closely affected by molecular crowding, has been neglected before, limiting our understanding of the role of cytoplasmic properties in physiological LLPS.^[^ ^7^ ^]^

Intracellular transport is crucial for biomolecule translocation and subcellular structure organization. In contrast to the in vitro environment, intracellular transport is complicated and consists of abnormal diffusive motion and active transport due to the nonequilibrium nature and complex cytoarchitecture of the cytoplasm.^[^ ^8^ ^]^ From a biophysical perspective, the dynamic behaviors of LLPS might be spatiotemporally associated with intracellular transport dynamics. For example, the assembly of molecules could be regarded as a diffusion‐reaction system.^[^ ^6^, ^9^ ^]^ In principle, the growth of the condensates is facilitated by the supply of new molecules diffusing from a distance, while the assembly would be aided by the locally slowed diffusion near the condensates; when the LLPS condensates grow to the extent approaching the mesh size of the cytoskeleton network (≈50 nm), their intracellular translocations and coalescences would rely on cytoskeleton‐associated active motion.^[^ ^10^ ^]^ In turn, LLPS in cells could also interact with intracellular dynamics by altering the cytoarchitecture and physical properties of the cytoplasm with emergent condensates and MLOs inside. Therefore, characterizing the complex intracellular dynamics during phase separation is essential to decipher the biophysical regulation of LLPS in living cells. Considering that LLPS has different processes over time and that the droplet size is gradually increasing, proper fluorescent tracers that can accurately quantify intracellular diffusion and active transport dynamics with long‐term spatiotemporal resolutions are needed. Quantum dots (QDs), as an emerging kind of fluorescent probe, have good biocompatibility, outstanding photostability, and moderate sizes and are a promising material for use in dynamic analysis in living cells.^[^ ^11^ ^]^

In this paper, by using a single‐particle tracking method, we spatiotemporally quantified both the diffusion dynamics of QDs and the active transport of endocytic fluorescent beads in cells undergoing SG assembly. These two kinds of movements resemble the diffusion for nanosized macromolecules and active motion for micron‐sized condensates. We found that both intracellular diffusion and active transport are slowed after the formation of SGs by LLPS. In addition, the formation of SG droplets altered the cytoplasmic environment by increasing spatial heterogeneity. More importantly, we demonstrated that the assembly of SGs is affected by intracellular diffusion rates and that the coalescence of SGs is aided by microtubule‐associated active transport. Our study not only reveals a close relationship between molecular crowding and intracellular phase separation but also suggests an important role of intracellular dynamics in regulating phase separation in vivo.

## 2 Result

**Figure 1:**
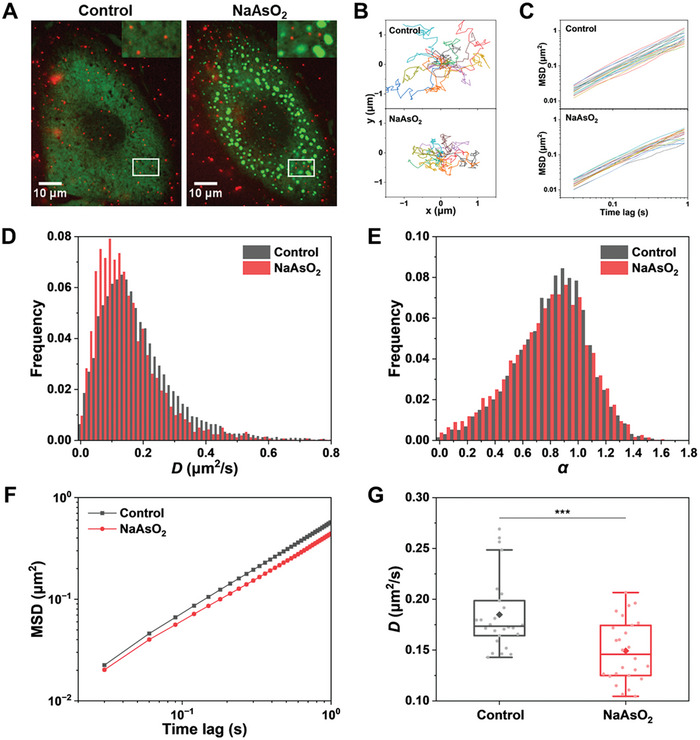
Intracellular diffusion is reduced with SG formation. A) Merged images of U2OS cells expressing EGFP‐G3BP1 (green) and loaded quantum dots (red). The cells were treated with 500 µm sodium arsenite (NaAsO_2_) for 30 min. Inset, enlarged view of the indicated area. B) Representative trajectories of diffusing QDs in control and NaAsO_2_‐treated cells. C) Mean square displacement (MSD) curves for the QD trajectories. D,E) Probability distributions of the diffusion coefficient *D* D) and exponent α E) of QD trajectories in control and NaAsO_2_‐treated cells. F) Comparison of the averaged MSD curves for QDs from different cells (*n* = 26). G) Comparison of diffusion coefficients *D* for QDs from different cells (*n* = 26). The boxes represent the interquartile range between the first and third quartiles, whereas the whiskers represent the 95% and 5% values, and the squares indicate the average. ^***^ *p *< 0.001.

We used the formation of stress granules (SGs) in human osteosarcoma cells (U2OS) expressing EGFP‐G3BP1 as the experimental model for studying intracellular LLPS.^[^ ^12^ ^]^ To measure the intracellular diffusion dynamics, we loaded quantum dots (QDs) into the cell cytosol through the osmotic lysis of pinocytic vesicles. These loaded QDs were individually dispersed and diffused in the cytoplasm, the trajectories of which provide a direct way to reliably quantify the diffusion behaviors of nanosized macromolecules.^[^ ^11^, ^13^ ^]^ To induce the assembly of SGs, sodium arsenite (NaAsO_2_) was added to the culture medium.^[^ ^12a,b^ ^]^ NaAsO_2_ can cause the phosphorylation of eukaryotic translation initiation factor 2α, which rapidly leads to translational arrest and dissociation of translation initiation complexes from polysomes. This results in the accumulation and assembly of stalled preinitiation translation complexes, including the RBPs and mRNAs, to form SGs, in which G3BP1 is a type of RBP.^[^ ^3^, ^4^ ^]^ Then, we monitored the diffusive behaviors of QDs during the LLPS process under a microscope (**Figure** **1**A). The trajectories of diffusing QDs in cells before and after SG formation were extracted (Figure 1B), and their mean square displacement (MSD) curves were calculated (Figure 1C). By fitting the MSD curves with MSD (*τ*) = 4*Dτ* ^α^ +*c*, we could determine the diffusion coefficient *D* and exponent α.^[^ ^14^ ^]^ The diffusion rate of QDs decreased after SG formation (Figure 1D), while the α (≈0.9) value was basically unchanged (Figure 1E). This suggests that the diffusive motion type of the QDs is not affected after the formation of SGs, but the diffusion rate was slowed, as was also illustrated by the shorter trajectories in SG‐formed cells (Figure 1B). Statistically, by analyzing 26 different cells with over 5000 trajectories, it was confirmed that diffusion was decreased in cells with formed SGs (Figure 1F,G). Moreover, the reduction in QD diffusion rates is comparable to the diffusion of QDs in dextran solutions with increased concentrations that adjust the molecular crowding effect, suggesting that the cytoplasm becomes more crowded after SG formation (Figure S1, Supporting Information). Additionally, to eliminate the possible effect of probe size and biochemical properties on intracellular diffusion, we used 70‐kD and 2000‐kD dextrans which are widely known nonspecific probes,^[^ ^11e,f^ ^]^ and the Qtracker which has a different surface coating from the QDs, respectively. All these probes show a reduction in the diffusion within cells during LLPS (Figure S2A–C, Supporting Information).

**Figure 2:**
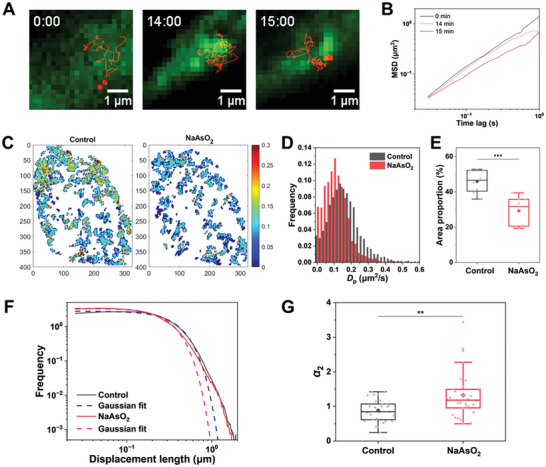
Spatial characteristics of intracellular diffusion during SG formation. A) Images of QD trajectory (red) overlaid with EGFP‐G3BP1 (green) in U2OS cells at different time points. The cells were treated with 500 µm NaAsO_2_ at 0 min. B) MSD curves of the diffusing QD shown in (A). C) Diffusion map for the U2OS cell shown in Figure 1A. D) Comparison of probability distributions of diffusion coefficients in the diffusion maps (*D*_P_) between the control and SG‐formed cells. E) Comparison of the occupied areas in the diffusion maps between control and SG‐formed cells. (*n* = 8, control; *n* = 8, NaAsO_2_). F) Probability density function (PDF) of the displacements for the QDs with or without NaAsO_2_ treatment. Dashed lines are the best fit for a Gaussian distribution. The time interval is 0.15 s. G) Non‐Gaussian parameter α_2_ for the QD displacement distributions with or without NaAsO_2_ treatment (*n* = 30, control; *n* = 30, NaAsO_2_). The boxes represent the interquartile range between the first and third quartiles, whereas the whiskers represent the 95% and 5% values, and the squares indicate the average. ^**^ *p *< 0.005, ^***^ *p *< 0.001.

The fluorescence images suggest that the formed SGs have obviously occupied the intracellular space (Figure 1A), which may alter the diffusive behaviors nearby. Indeed, this hypothesis is confirmed by the typical movement of diffusing QDs during the initial period of SG formation (**Figure** **2**A). When SGs began to form, the QDs appeared to collide with the SG droplets and became impeded and even temporally compartmentalized between the SG droplets. The MSD curves for the QD gradually declined, consistent with our observations (Figure 2B). Obviously, SG formation has added to heterogeneity in the cytoplasm. It is also observed that the stress granules are not accessible for the diffusing QDs, which was further verified by quantifying the colocalization between the SGs and QDs (Figure S3, Supporting Information). Next, to map the spatial diffusion characteristics across the whole cell, we plotted the diffusion map and exponent α map of the cell by dividing the cells into square grids and calculating the local diffusion coefficients of each grid node from the trajectory segments near the node (Figure 2C; Figure S4A,B, Supporting Information).^[^ ^11e^ ^]^ The diffusion maps show that after SG formation, the diffusion rate decreased across the cell, as expected (Figure 2D). The distribution of α from the diffusion map becomes slightly broader after SG formation, suggesting that the diffusion modes of QDs become more diverse (Figure S4C, Supporting Information). This could be contributed to the increased intracellular heterogeneity, as illustrated by the broadened fluorescence intensity distribution for all the points in the SG‐formed cell (Figure S4D, Supporting Information). More importantly, more empty areas appeared after the formation of SGs (Figure 2E), indicating that the diffusing space for QDs was reduced.

To further characterize the intracellular QD diffusion dynamics, we analyzed the probability density function (PDF) of the displacements for the QDs.^[^ ^14^, ^15^ ^]^ For normal diffusion in a homogeneous environment, the diffusion exhibits a Gaussian distribution of displacements. For diffusion in the cytoplasm, the QDs diffusing in cells would have a divergence from the Gaussian distribution due to cytoplasmic heterogeneity. Interestingly, we noticed that the QDs have a stronger divergence (Figure 2F). To quantify the deviation from the Gaussian distribution, we calculated the non‐Gaussian parameter α_2_ by α_2_ = $\;\frac{\left\langle dx^{4}\right\rangle }{2{\left\langle dx^{2}\right\rangle }^{2}}-1$. The α_2_ value at 0 is an ideal Gaussian distribution and increases with the degree of deviation.^[^ ^14^, ^15^ ^]^ Our results show that α_2_ increases after LLPS (Figure 2G), suggesting that the cytoplasmic environment where QDs diffuse becomes more heterogeneous due to SG droplets.

**Figure 3:**
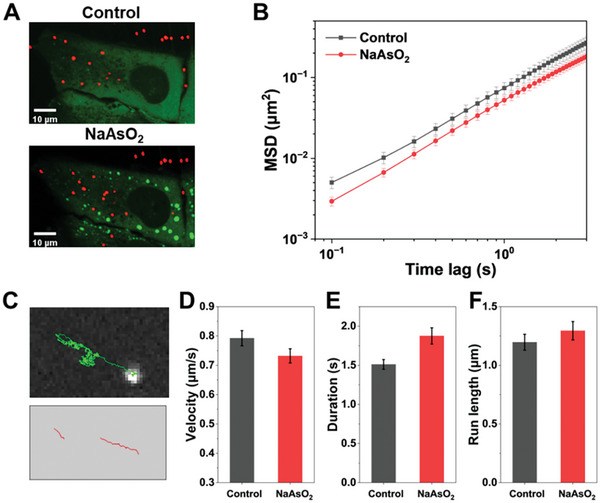
The directed motion of intracellular vesicles is slowed in SG‐formed cells. A) Merged images of U2OS cells expressing EGFP‐G3BP1 (green) with endocytosed 500‐nm fluorescent microspheres (red). The cells were treated with 500 µm NaAsO_2_ for 30 min to induce SG assembly. B) Averaged MSD for all trajectories of fluorescent microspheres as a function of lag time. (*n* = 74, control; *n* = 69, NaAsO_2_). C) Representative trajectory of endocytic 500‐nm fluorescent beads (upper panel) and segments of directed motion detected from the trajectory (lower panel). D–F) Average velocity D), duration E), and run length F) for the directed motion in control and NaAsO_2_‐treated cells. (*n* = 238, control; *n* = 246, NaAsO_2_). Error bars indicate SEM.

The intracellular transport behavior is also correlated with the particle size. When the cargo is over the mesh size of the actin network, it is constrained by the cytoskeleton and transported under intracellular active forces. To measure the active transport of large probes in cells under LLPS, 500‐nm fluorescent beads were endocytosed to label the transport of endocytic vesicles (**Figure** **3**A).^[^ ^16^ ^]^ After the induction of SGs by NaAsO_2_, we found that the movements of endocytic vesicles were slowed (Figure 3B). Similar results were also observed when using the 100‐nm fluorescent beads (Figure S2D, Supporting Information). Furthermore, we examined whether the dynamics of directed transport are also altered in LLPS cells by using an automatic algorithm to identify and extract directed motion segments from all trajectories (Figure 3C).^[^ ^17^ ^]^ Interestingly, we found that in cells with SGs, the velocities of directed motion decrease, while the duration increases (Figure 3D–F), which is similar to a previous measurement for motor proteins in crowded media. Together, with the above results for QD diffusion, it is shown that the diffusion of nanosized probes as well as the active transport of micron‐sized vesicles are both reduced in cells with SG assembly, suggesting that molecular crowding may play roles in regulating intracellular dynamics under LLPS.

**Figure 4:**
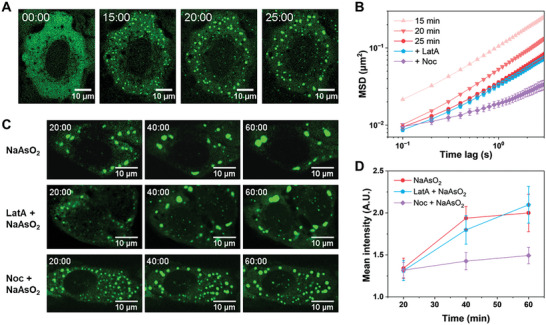
The intracellular transport of SGs and its correlation with the cytoskeleton. A) Fluorescence images of U2OS cells expressing EGFP‐G3BP1 (green) at different times treated with 500 µm NaAsO_2_. Time, minute: second. B) Averaged whole MSD for all trajectories of the SG droplets under different conditions as a function of lag time. SGs were induced by 500 µm NaAsO_2_ and then imaged at the indicated time points. Latrunculin (LatA) or nocodazole (Noc) was added to the cells after 25 min when SGs had already formed. C) Fluorescence images of U2OS cells expressing EGFP‐G3BP1 under different conditions. Before imaging, the cells were pretreated with LatA for 10 min or Noc for 20 min to destroy the actin filaments or microtubules, respectively. Then, the cells were treated with 500 µm NaAsO_2_ to induce the LLPS of SGs and imaged at the indicated time points. D) Changes in the mean fluorescence intensities for intracellular SG droplets with time. NaAsO_2_ (*n* = 13), LatA and NaAsO_2_ (*n* = 12), Noc and NaAsO_2_ (*n* = 13). Error bars indicate SEM.

Next, we sought to investigate the transport dynamics of SGs since they increase in size during LLPS and are expected to be influenced by the altered environment of the cytoplasm. By tracking the LLPS droplets, the MSD results show that the movements of SGs are gradually slowed down with the progression of LLPS (**Figure** **4**A,B), which is likely attributed to the enlarged size of SGs. With the coalescence of SG droplets, the fluorescence intensity of SG droplets gradually increases (Figure S5, Supporting Information). In addition, we compared the diffusion rates of newly assembled condensates with similar fluorescent intensities and found similarly reduced diffusion rates (Figure S5, Supporting Information). This result suggests a gradually enhanced molecule crowding with the LLPS process, which is consistent with the results for QDs and endocytic beads. Moreover, no obviously directed motion could be detected for SGs compared with that for endocytic beads (Figure S6, Supporting Information). However, we notice that SGs are still very mobile at 25 min even when large condensates have formed, attracting us to explore the mechanism of the intracellular transport of SGs. To this end, we used latrunculin (LatA) and nocodazole (Noc) to disrupt actin filaments and microtubules, respectively. Interestingly, we found that Noc treatment obviously impeded SG movement (Figure 4B). Microtubules contribute to the intracellular transport of MLOs, which is possible because of the nonspecific adhesions between them.^[^ ^10^, ^18^ ^]^ The correlation between SG dynamics and microtubules inspired us to explore the role of active transport in LLPS. To this end, we first disrupted the cytoskeleton and then induced SGs with NaAsO_2_. Actin destruction did not affect the LLPS of SGs; however, microtubule destruction obviously hindered the coalescence of SG droplets (Figure 4C,D). Our results suggest that the active transport of micron‐sized droplets accelerates the coalescence of LLPS, which is distinct from the in vitro situation with no active transport, and the diffusion of large droplets would be slowed down due to the Stokes–Einstein dependence on particle sizes.

**Figure 5:**
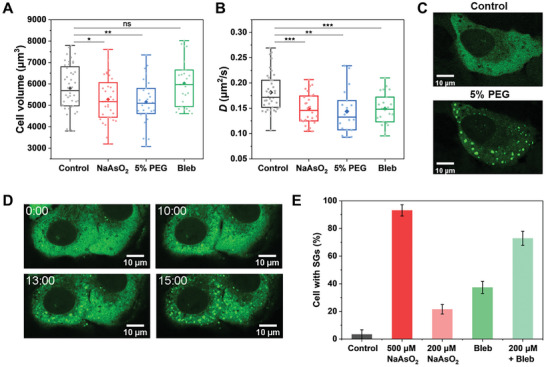
Effect of molecular crowding and intracellular diffusion on SG formation. A,B) Cell volume A) and diffusion coefficient B) under different conditions. Control group (*n* = 44), 500 µm NaAsO_2_ treatment (*n* = 32), 5% PEG 300 treatment (*n* = 27), and Bleb treatment (*n* = 25). The boxes represent the interquartile range between the first and third quartiles, whereas the whiskers represent the 95% and 5% values, and the squares indicate the average. ^*^ *p *< 0.05, ^**^ *p *< 0.005, ^***^ *p *< 0.001, ns, not significant. C) Fluorescence images of U2OS cells expressing EGFP‐G3BP1 (green) treated with 5% PEG 300 for 30 min. D) Fluorescence images of U2OS cells expressing EGFP‐G3BP1 (green). The cells were treated with 100 nm Bleb at 0 min. E) Percentage of cells with SGs when treated with 500 or 200 µm NaAsO_2_, or with 100 nm Bleb, or with 200 µm NaAsO_2_ and 100 nm Bleb. (*n* = 84, control; *n* = 91, 500 µm NaAsO_2_; *n* = 123, 200 µm NaAsO_2_; *n* = 133, 100 µm Bleb; *n* = 127, 200 µm NaAsO_2_ and 100 nm Bleb). Error bars indicate SEM.

The intracellular transport dynamics are regulated by the physical properties of the cytoplasm, in which molecular crowding is known to play a key role. To check whether the molecule crowding is altered in the NaAsO_2_‐treated cells, as under hypertonic stress, we measured the cell volume and found that NaAsO_2_ indeed leads to a slight decrease in the cell volume by ≈8% (**Figure** **5**A), which agrees with the reduced intracellular diffusion of QDs we observed (Figure 5B). These results support the hypothesis for molecular crowding. Nevertheless, we sought to check whether the reduction in cell volume would result in both similarly decreased intracellular transport and phase separation of SGs. To this end, we added 5% PEG 300 to the culture medium to increase the external osmotic pressure; the cell volume decreased to a similar extent as that in NaAsO_2_‐treated cells (Figure 5A). We found that the phase separation of SGs takes place in cells under such hypertonic stress after 15 min, in agreement with a previous report (Figure 5C). Moreover, the diffusion of QDs in these cells decreased accordingly (Figure 5B). The dynamics of directed motion also show similar changes as those in NaAsO_2_‐treated cells (Figure S7, Supporting Information). Together, these results demonstrate the correlation between the decrease in cell volume, the reduced intracellular transport dynamics, and the phase separation of SGs, suggesting that the increased molecular crowding also plays a roles in LLPS in response to oxidative stress by NaAsO_2_.

The enhanced molecular crowding as a result of cell volume shrinkage has increased the molecular concentration and simultaneously reduced the diffusion rates, which the former is considered to be critical for promoting LLPS. To investigate the role of intracellular dynamics in the LLPS of SGs, we sought to tune the intracellular diffusion rates without shrinking the cell volume. To this end, we treated cells with blebbistatin (Bleb) to inhibit actomyosin contractions and slow active fluctuations in the cells. As expected, the diffusion of QDs is shown to be reduced while the cell volume is slightly increased, which is consistent with previous results (Figure 5A,B).^[^ ^19^ ^]^ Strikingly, we found that some cells had newly formed SGs within 10 min after adding Bleb alone (Figure 5D). Since Bleb may have phototoxicity under long‐term fluorescence imaging, we only imaged the cells once after the cells were treated with Bleb to avoid other possible influencing factors. We also confirmed that no SG would be formed under continuous laser excitation if no drug was added (Figure S8, Supporting Information). Statistical results show that 40% of cells have SGs with only Bleb treatment. Although the percentage did not reach the values with 500 µm NaAsO_2_ treatment, it was significantly greater than that of the control group, suggesting a newly promoting effect of Bleb treatment on LLPS. Furthermore, we found that compared with treatment with 200 µm NaAsO_2_, in which ≈20% of cells contained SGs, the addition of Bleb greatly enhanced the percentage to over 70% of cells (Figure 5E). These results suggest that the reduced diffusion rate facilitates the LLPS of SGs in cells.

## 3 Discussion

In this paper, we investigated the complex intracellular transport dynamics in cells undergoing phase separation by using single‐particle tracking. We found that after the formation of SGs, both the intracellular diffusion of QDs and the active trafficking of endocytic fluorescent beads are reduced, suggesting an enhanced crowding environment inside the cells undergoing LLPS. Molecule crowding is widely used to advance in vitro LLPS experiments and recently exhibited similar regulatory roles in LLPS within living cells under volumetric compression.^[^ ^5^, ^6^, ^20^ ^]^ Here, by combining intracellular dynamics characterization with cell volume measurement, our study shows that the upregulation of molecular crowding occurs not only under hyperosmotic compression but also in response to hyperoxidation, such as NaAsO_2_. These results suggest that the extent of molecular crowding is generally regulated in LLPS in cells, supporting the existing investigations of in vitro LLPS on the crowding effect.^[^ ^5a,d,e^ ^]^ Moreover, the mechanism of molecular crowding on LLPS phase separation remains unclear. Previously, it was considered that molecular crowding promotes LLPS by increasing molecular concentration. However, the impact of diffusion dynamics on LLPS has generally been disregarded. The new finding that the reduced diffusion caused by intracellular molecular crowding also contributes to the assembly of SGs, improves our understanding of the role of molecular crowding on LLPS. Nevertheless, we should note that the excluded volume effect on LLPS resulting from crowding has not yet been determined. In the complex cytoplasm, these parameters intertwine with each other, and their contributions are difficult to decouple, which requires well‐designed experimental strategies.

More importantly, for the first time, we have demonstrated that SG formation could be regulated by intracellular diffusion and active transport in multiple ways. Our results have shown that reducing intracellular diffusion without shrinking cell volumes could promote the assembly of SGs and that disrupting microtubule‐associated transport could hinder the coalescences of SG droplets.^[^ ^10b^ ^]^ These results reveal the complex role of intracellular nonequilibrium dynamics in the spatiotemporal assembly and organization of MLOs, which is obviously distinct from the in vitro environments. Moreover, using the diffusion map and the distribution of displacements of quantum dots in cells, it is shown that the intracellular environment is altered by LLPS as well, becoming more heterogeneous and compartmentalized due to emerging MLOs. As such, considering that intracellular dynamics are closely affected by intracellular environments, the existence of an interplay between intracellular dynamics and LLPS should be acknowledged.

The association between intracellular diffusion and SG assembly provides new evidence for the idea that LLPS could be regarded as a diffusion‐reaction system. From the view of intracellular dynamics, our study sheds new light on the understanding of phase separation phenomena. In yeast cells, glucose starvation was reported to reduce intracellular diffusion, which would facilitate the formation of filament structures.^[^ ^21^ ^]^ The number of ribosomes could alter the extent of molecular crowding and thus modulate the intracellular diffusion rates, which controls the phase separation in cells.^[^ ^22^ ^]^ Intracellular dynamics may provide a new approach to regulate intracellular phase separation and help the treatment of LLPS‐related diseases.

## 4 Experimental Section

### Cell Culture and Drug Treatment

Human osteosarcoma U2OS cells were maintained in Dulbecco's modified Eagle medium (DMEM, GIBCO) with 10% fetal bovine serum (FBS, GIBCO) and 1% penicillin‒streptomycin (GIBCO) and incubated at 37 °C with 5% CO_2_. Cells at the log phase were seeded at the bottom of Petri dishes the day before the experiments. Chemicals were from Sigma‒Aldrich unless otherwise stated.

To induce the formation of stress granules, the cells were incubated with 500 µm sodium arsenite (NaAsO_2_). To disrupt the microtubules or actin filaments, the cells were incubated with 20 µm nocodazole (Noc) for 30 min or with 100 nm latrunculin A (LatA) for 10 min, respectively. To inhibit myosin activity, the cells were incubated with 100 nm blebbistatin (Bleb) for the indicated times. To increase the external osmotic pressure, the cells were incubated with 5% PEG 300 for 10 min. These drugs were maintained in the medium throughout the experiments.

### Plasmid Construction and Stable Cell Line Construction

The plasmids used were created by standard molecular biology techniques and confirmed by exhaustively sequencing the cloned fragments. In particular, for pLVX‐EF1α‐EGFP‐G3BP1, we inserted EGFP‐G3BP1 downstream of the EF1α promoter with a ClonExpress Ultra One Step Cloning Kit (C115‐01, Vazyme). The EGFP‐G3BP1 sequence was cloned from pEGFP‐C1‐G3BP1‐WT (135 997, Addgene). The construction of a stable cell line by lentivirus infection and all plasmids used for lentivirus packaging were prepared by an E.Z.N.A. Endo‐free Plasmid DNA Mini Kit (D6950‐01B, Omega). Lentivirus packaging by cotransfecting with psPAX2 (12 260, Addgene), pMD2. G (12 259, Addgene) and pLVX‐EF1α‐EGFP‐G3BP1 into HEK293T cells at a ratio of 3:1:4 (5 µg in total) in 6 cm dishes, and the viral supernatant was collected at 48 h after transfection and filtered through a 0.45 µm PES filter to remove cell debris. U2OS cells were infected with filtered viral supernatant, and 72 h after infection, EGFP‐positive cells were collected by cell sorting.

### Fluorescent Probes and Internalization

Quantum dots were semiconductor nanoparticles made from a CdSe core and ZnS shell and were chemically coated to minimize nonspecific interactions. The QD (Q10123MP, Invitrogen) was coated with PEG and streptavidin, with a size of ≈29 nm and a weak negative charge; the Qtracker (Q21021MP, Invitrogen) was coated with PEG, with a smaller size than the QD as it had no streptavidin coating and a neutral charge. Both QDs have an emission at 655 nm. Fluorescent beads were polystyrene latex nanoparticles with indicated sizes and chemical modifications. The 100‐nm bead (L9904, Sigma) was amine‐modified and thus positively charged; the 500‐nm bead (L3280, Sigma) was carboxylate‐modified and thus negatively charged. Dextrans were well‐known probes without nonspecific interactions with the cytoplasm. The 70‐kD rhodamine B‐dextrans (D1841, Invitrogen) and 2000‐kD TRITC‐dextrans (D7139, Invitrogen) were used, which were both neutral.

To measure the intracellular diffusion dynamics, the small probes (dextrans, QDs) were loaded into cells through the osmotic lysis of pinocytic vesicles (I‐14402, Invitrogen). First, the probes were mixed with a hypertonic solution at a concentration of 0.5−5 nm QDs or 0.2−0.5 mg mL^−1^ dextrans. Cells were incubated in a solution at 37 °C for 15 min to allow the probes to be carried into the cells via pinocytic vesicles. Then, the cells were transferred to a hypotonic medium for 2 min, which caused the release of trapped QDs from the pinocytic vesicles into the cytoplasm. After that, the cells were placed in complete DMEM at 37 °C for 15 min to recover. Before imaging, the cells were washed three times using DMEM. To measure the intracellular active transport, the fluorescent beads were endocytosed into cells. The fluorescent beads were mixed with DMEM, and the cells were incubated in this mixed solution for 3 h to allow the beads to enter the cells. Then, the cells were washed with DMEM three times before imaging.

### Fluorescence Microscopy

Fluorescence images and movie files were obtained using microscope (Olympus IX73) equipment with total internal reflection fluorescence (TIRF) illumination and spinning‐disk confocal imaging mode (Yokogawa W1). A 60× oil‐immersed objective (1.50 N.A., Olympus) and a back‐illuminated EMCCD camera (Andor iXon Life 888) were used. The pixel size is 0.2166 µm. To maintain cell physiology, an on‐stage incubator (OKO Lab) was installed on the microscope to maintain 37 °C and 5% CO_2_ for cells during imaging. To track the movements of fluorescent particles, highly inclined and laminated optical sheet (HILO) imaging was performed by properly decreasing the incident angle of the laser. A 561 nm laser was used to excite the QD, 70‐kD, and 2000‐kD dextrans, Qtracker, 100 and 500‐nm fluorescence beads. The movements of QDs and 2000‐kD dextrans in cells were acquired as movie files with a total of 1 min at 30 ms intervals, the 70‐kD dextrans were recorded with a total of 30 s at 10 ms intervals, and the endocytic fluorescence beads were recorded with a total of 3 min at 100 ms intervals. To track the movements of SGs, the spinning‐disk confocal mode was applied, with recording for 3 min at 100 ms intervals. A 488 nm laser was used to excite EGFP‐G3BP1.

### Single Particle Tracking

The Particle Tracker plug‐in in Image was used to track individual particles from the videos. First adjusted the parameters of the radius, cutoff, and percentile for each frame to distinguish the particles from the background. In the linking process, the linking range and displacement parameters were adjusted to realize the connection of particle positions between frames. After that, all detections and connections were visually checked, and trajectories over 30 frames were selected for further analysis.

### Data Analysis

Dynamical analysis of the trajectories was performed using a user‐defined program in MATLAB. Before MSD analysis of QDs, all trajectories were filtered twice: 1) intensity filtering, removing the QDs whose intensities were more than 1.5 times the average intensity of all QDs and less than 0.5 times the average intensity, to remove potential aggregates and out‐of‐focus QDs; 2) immobile particle filtering, removing QDs with displacements less than 0.2 µm. In the case of dextrans, fluorescence beads, and SG droplets, the corresponding procedures and parameters were adjusted. The time and ensemble‐averaged mean square displacement (MSD), <Δr^2^(τ) >, where Δr(τ) = r (t+τ) – r (t), τ is the time lag. The diffusion coefficient (*D*) is determined by linear fitting of the first three points from the MSD, MSD (τ) = 4*D*τ^α^ + c. The exponent α is the slope of the MSD curve in the log‐log plot, which contains information about the motion modes.^[38]^ The value of the exponent α is between 0 and 2, in which an α of 1 indicates Brownian motion and an α <1 indicates subdiffusion and confined motion.

### Diffusion Map Plotting

To analyze the spatial dynamics of QDs, the plotting of spatial maps (spatial diffusion coefficient *D*_P_ and exponent α_p_) of QD dynamics on a cell was achieved using a user‐defined algorithm in MATLAB. Briefly, the area of the cell was divided into square grids. Each grid point was the unit of the diffusion map, and the grid size corresponded to the spatial resolution of the map. In the experiment, the grid size was chosen to be three pixels, and the distance threshold was set to 2.5 pixels by considering the diffusion rate of QDs. To calculate the spatial dynamics at each grid point, spatially local trajectory segments were first selected within a distance threshold to the grid point. Then, the spatially local MSD for each grid point was calculated using these segments separately. Next, the *D*_P_ and α_p_ at each grid point were determined by fitting the spatially local MSD curves with the equations mentioned above. Finally, the contour maps of the *D*_P_ and α_p_ were plotted in MATLAB using a built‐in smoothing process. The blank regions or white points in these maps indicated that there was not sufficient QD trajectory for calculating the local diffusion dynamics.

### Cell Volume Measurements

Cells were labeled with CellTracker Green CMFDA (C7025, Invitrogen) and observed under a 60× oil‐immersed objective (1.50 N.A., Olympus) with optical cross‐sections at 0.3 µm *z*‐axis intervals. Cells were randomly selected. 3D visualization was carried out by using ImageJ and Imaris software. The cell volume was calculated by counting the effective voxel number. A threshold was chosen as the fluorescence intensity on the bottom interface of the cell, which could be identified from the reflection signal, which was confirmed as the intensity defining the boundary on the x‐y plane fluorescence intensity map. The z‐stack images were segmented, and the cellular volumes were computed using custom MATLAB scripts.

### Statistical Analysis

For comparison, a two‐sided Student's *t*‐test was applied using the Origin software. A two‐tailed Student's *t*‐test was used for comparisons between the two groups. ^*^ *p* < 0.05, ^**^ *p *< 0.005, ^***^ *p *< 0.001, ns, not significant. All the measurements were taken in more than three independent experiments. In box plots, the boxes represent the interquartile range between the first and third quartiles, whereas the whiskers represent the 95% and 5% values, and the squares indicate the average.

## Conflict of Interest

The authors declare no conflict of interest.

## Supporting information

Supporting Information

## Data Availability

The data that support the findings of this study are available from the corresponding author upon reasonable request.
