# Supplementary material for: Interplay Between Intracellular Transport Dynamics and Liquid‒Liquid Phase Separation
Source: Adv Sci (Weinh). 2024 Mar 6;11(19):2308338. doi: 10.1002/advs.202308338 (PMC11109639; doi:10.1002/advs.202308338)
Supplement: Supplementary file 1 — Supporting Information [file ADVS-11-2308338-s001.pdf]

ADVANCED SCIENCE
Open Access

Supporting Information

for *Adv. Sci.*, DOI 10.1002/advs.202308338

Interplay Between Intracellular Transport Dynamics and Liquid–Liquid Phase Separation

Ming-Li Zhang, Ziheng Zhang, Xue-Zhi Niu, Hui-Ying Ti, Yu-Xuan Zhou, Bo Gao, Yiwei Li, Ji-Long Liu, Xiaosong Chen and Hui Li**

Supporting Information

Interplay between intracellular transport dynamics and liquid–liquid phase separation

Ming-Li Zhang[1], *Ziheng Zhang*[2], *Xue-Zhi Niu*[1], *Hui-Ying Ti*[1], *Yu-Xuan Zhou*[1], *Bo Gao*[1], *Yiwei Li*[3], *Ji-Long Liu*[2,4], *Xiaosong Chen**[1], *Hui Li**[1]

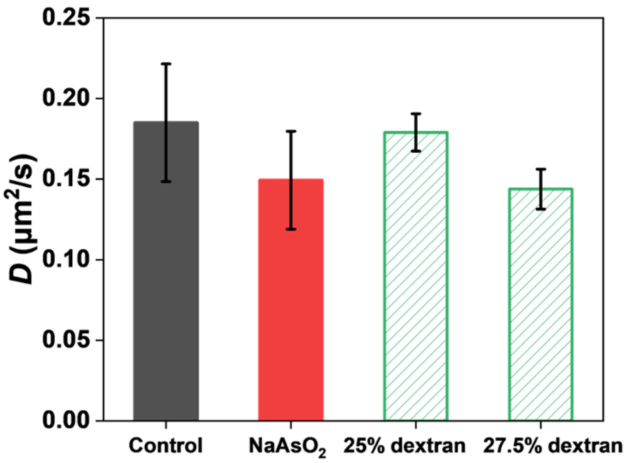

Figure S1. Comparison of diffusion coefficients D for QDs in cells and in 10-kD dextran solutions. Cell number, control (n =26); $NaAsO_2$ (n = 26); Sample number, 25% dextran (n = 8); 27.5% dextran (n = 8). Error bars indicate SEM.

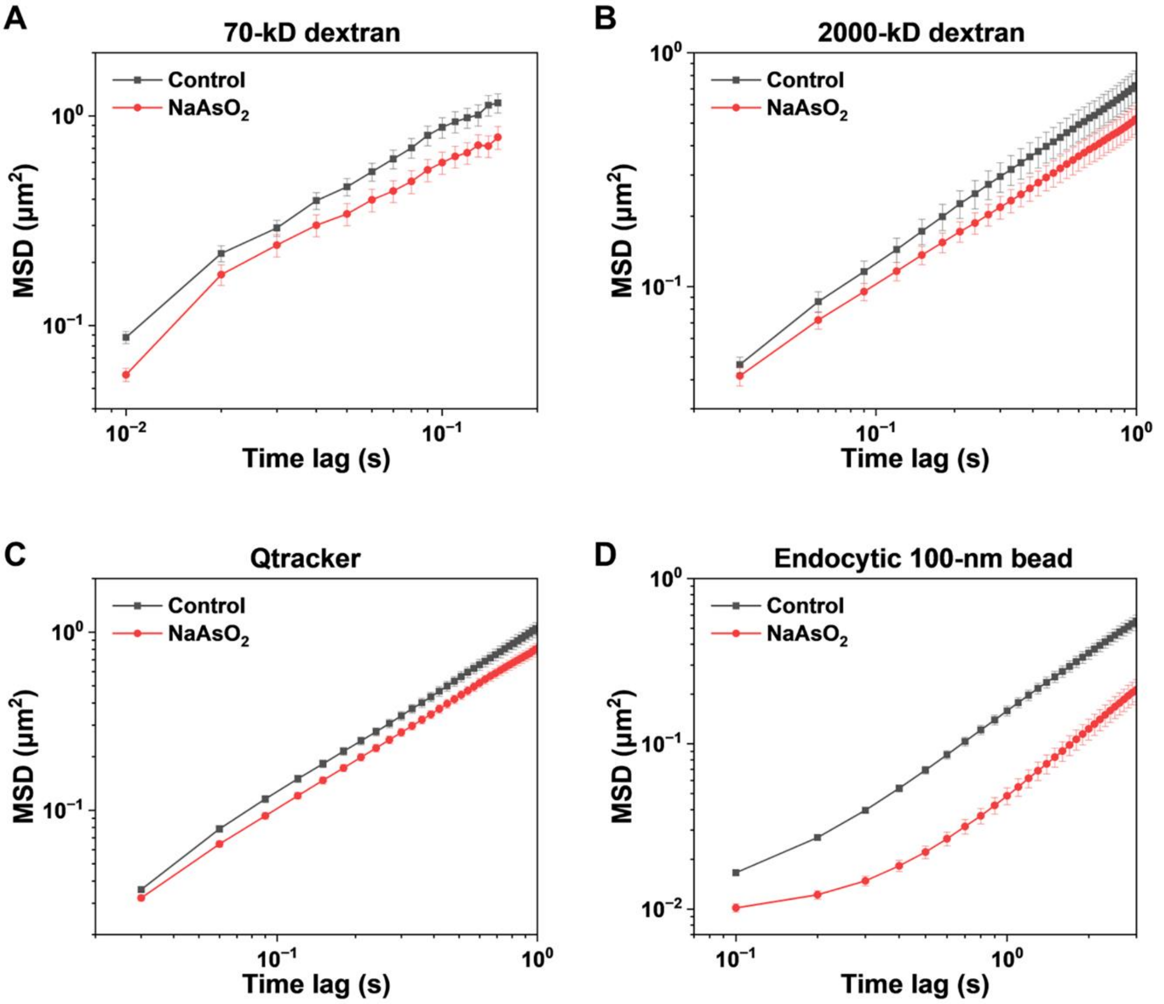

Figure S2. Comparison of the averaged MSD curves for 70-kD dextrans (A), 2000-kD dextrans (B), Qtracker (C), and endocytic 100-nm fluorescent beads (D) in control and SG-formed cells. Cell numbers, 70-kD dextrans (n = 16); 2000-kD dextrans (n = 11); Qtracker (n = 10); 100-nm bead (n = 8). Error bars indicate SEM.

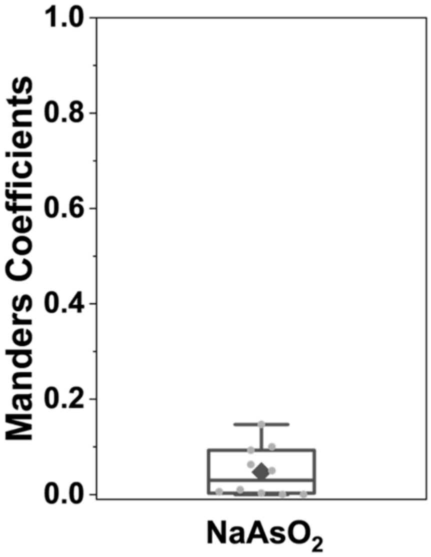

Figure S3. Quantification of the colocalization between QDs and SGs in cells by the Manders coefficients. The Manders coefficients were calculated as the fraction of total QD fluorescence intensities that colocalizes with the fluorescence of SGs. The averaged Manders coefficient is measured to be 0.0472, indicating that there is almost no QDs overlap the SGs. The boxes represent the interquartile range between the first and third quartiles, whereas the whiskers represent the 95% and 5% values, and the squares indicate the average. Cell number, n=10.

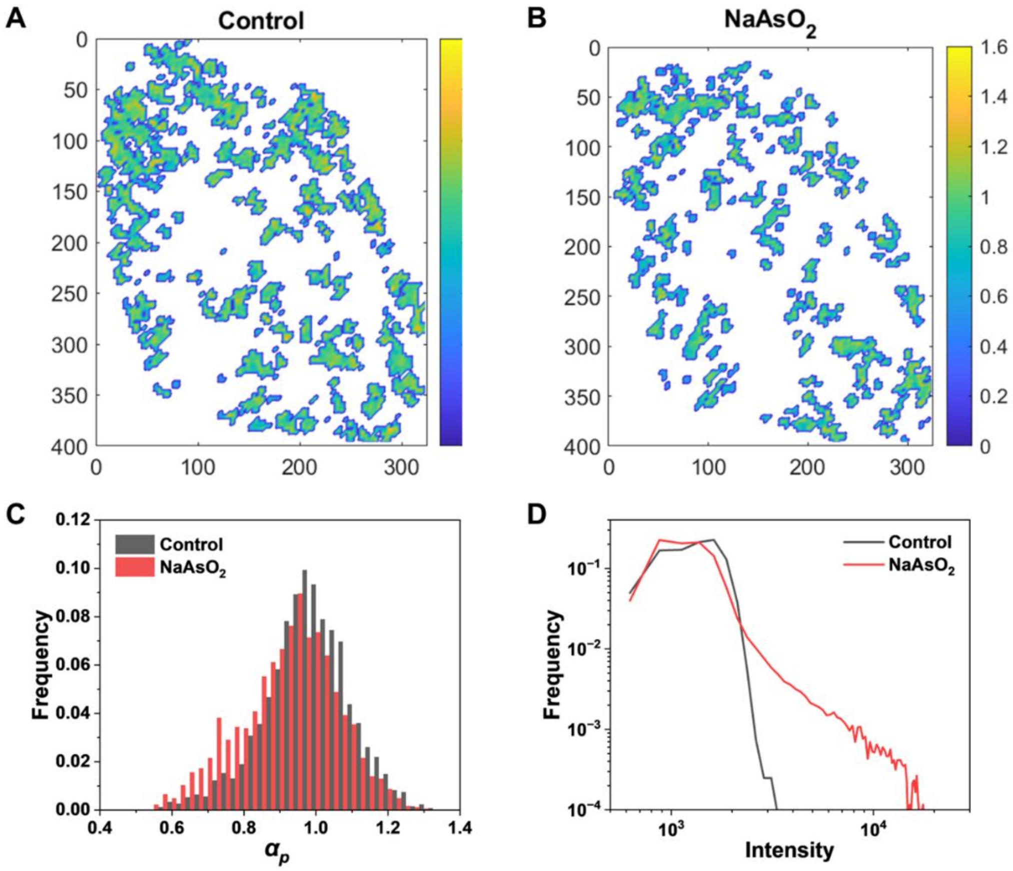

Figure S4. Spatial heterogeneity of intracellular diffusion during SG formation. A.B) Exponent α map for the U2OS cell in Figure 1a. C) Comparison of probability distributions of α in the diffusion maps (α_P) between the control and SG-formed cells. D) Comparison of probability distributions of intracellular fluorescence intensity for all the pixels in a cell.

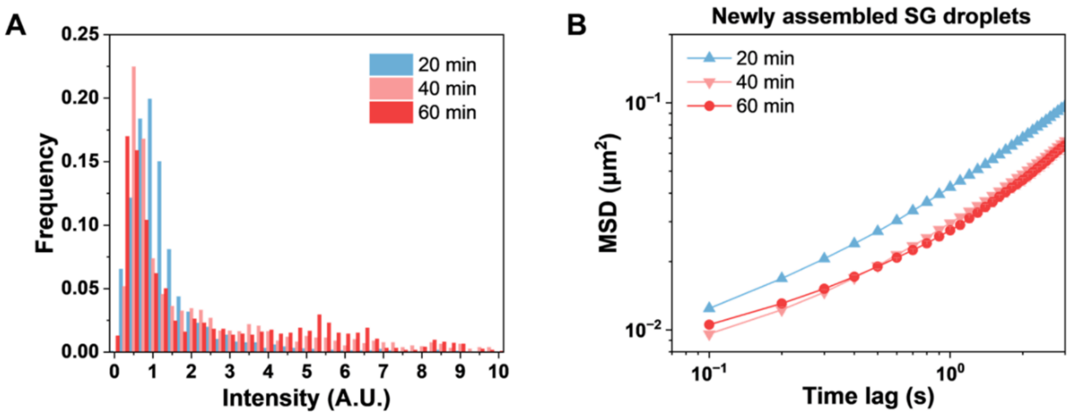

Figure S5. The changes of fluorescence intensities and MSD for SG droplets. SGs were induced by 500 μM $NaAsO_2$ and then imaged at the indicated time points. A) Comparison of probability distributions of fluorescence intensity of intracellular SG droplets. It is shown that the fluorescence intensity of SG droplets gradually increases with time, due to the coalescences of SG droplets. B) Comparison of the averaged MSD for newly assembled SG droplets. Only the droplets with the fluorescent intensities smaller 1 were analyzed.

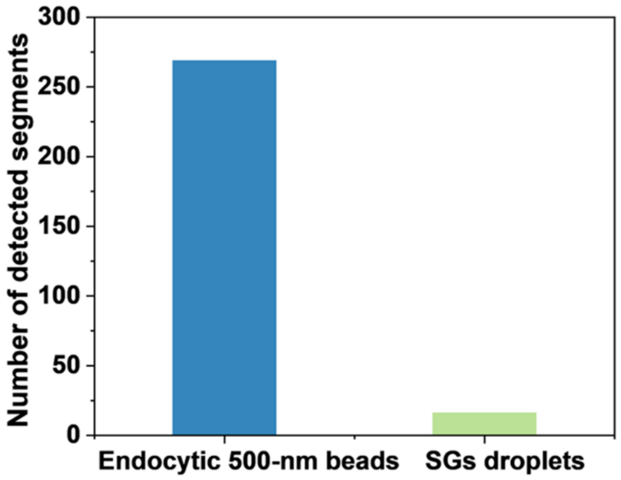

Figure S6. Comparison of the number of detected motion segments. The number of detected motion segments of all trajectories in endocytic 500-nm beads (n = 305) and SG droplets (n = 325).

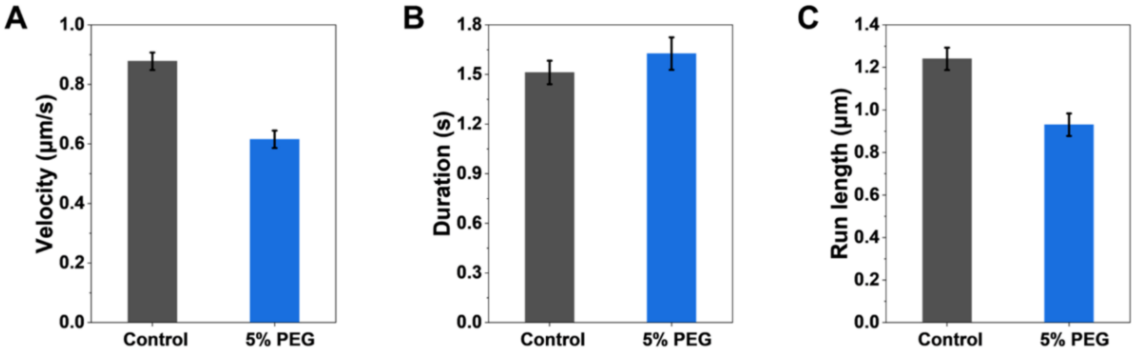

Figure S7. The dynamics of directed motion in 5% PEG 300 - treated cells. A-C) Average velocity (A), duration (B), and run length (C) for the directed motion in control and 5% PEG 300-treated cells. (n = 232, control; n = 113, 5% PEG 300). Error bars indicate SEM.

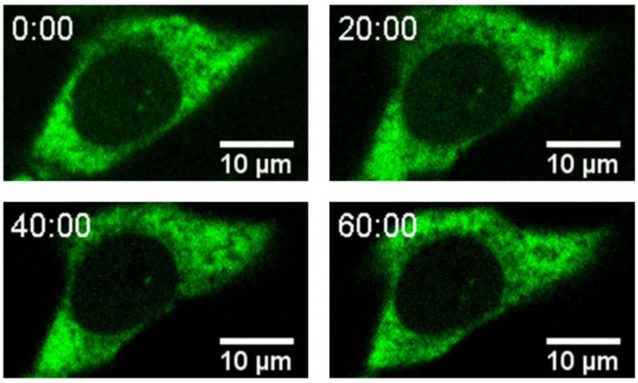

Figure S8. Fluorescence images of control group. Fluorescence images of U2OS cells expressing EGFP-G3BP1 (green) at different times without any treatment.
